# Draft Genome Sequence of *Novosphingobium panipatense* Strain P5:ABC, Isolated from Hydrocarbon-Contaminated Soil from Noonmati Refinery, Assam, India

**DOI:** 10.1128/genomeA.01265-17

**Published:** 2017-11-09

**Authors:** Arvind Kumar Singh, Bobby Chettri, Arpita Ghosh, Surendra K. Chikara, Timir Tripathi

**Affiliations:** a Department of Biochemistry, North Eastern Hill University, Shillong, India; b Eurofins Genomics, Doddanakundi Industrial Area 2, Seetharampalya, Hoodi, Bengaluru, Karnataka, India

## Abstract

*Novosphingobium panipatense* P5:ABC is a hydrocarbon-degrading bacterium isolated from petroleum-contaminated soil. Here, we present the 5.74-Mb draft genome sequence with 5,206 genes and an average G+C content of 64.7%. The genomic information will improve our understanding of the diversity of *N. panipatense* and the mechanisms of microbe-based hydrocarbon degradation.

## GENOME ANNOUNCEMENT

Genus *Novosphingobium* is a yellow pigmented, strictly aerobic, non-spore-forming, rod-shaped, Gram-negative bacterium. It has been isolated from sediments contaminated with crude oil (1, 2), pesticides (3, 4), and freshwater sediments (5). Members of this genus have the capacity to degrade aromatic compounds (6–8). Currently, only three complete genome sequences of aromatic hydrocarbon-degrading *Novosphingobium* spp. are published (9–11). We have isolated a bacterial species from crude oil-contaminated sediment collected from the Noonmati Refinery, Assam, India. The isolate utilizes both aliphatic and aromatic hydrocarbons as sole sources of carbon and energy and was named as *N. panipatense* P5:ABC based on its 16S rDNA-based similarity with *N. panipatense*, initially isolated from an oil-contaminated site in Panipat, India (1). Here, we report the complete genome sequence of isolate *N. panipatense* P5:ABC.

The genomic DNA from *N. panipatense* P5:ABC was isolated to generate 670 Mb of data with 150 bp paired-end (PE) chemistry using Illumina NextSeq 500. The assembly was carried out using Velvet assembler v1.2.10 (12). The total assembled genome is 5.74 Mb with 99 scaffolds. The *N*_50_ value and average length are 181,126 bp and 57,930 bp, respectively. The genome is estimated to have an overall G+C content of 64.7%. The machine learning algorithm based Prodigal v2.50 tool predicted 5,206 genes, where lengths of genes range from 23 to 3,828 amino acids (13). Blast2Go was used to annotate the predicted genes.

Information about the genome sequence of *N. panipatense* P5:ABC will provide insight into the diversity and mechanisms of hydrocarbon degradation in the environment. During our analysis, we found the presence of several hydrocarbon-degrading and related genes, namely, dioxygenases, rubredoxin, esterase, alkane-1-monooxygenase, alkane sulfonate monooxygenase, formate dehydogenase, 4-carboxymuconolactone decarboxylase, 3-carboxy-*cis*, *cis*-muconatecycloisomerase, 3-oxoadipate-enol-lactonase, protocatechuate-3, and salicylaldehyde dehydrogenase (14–16). This knowledge of the gene might be used to improve the bioremediation technologies used in this organism.

### Accession number(s).

This whole-genome shotgun project of *Novosphingobium panipatense* P5:ABC has been deposited at DDBJ/ENA/GenBank under the accession number MSQB00000000.
